# Early behavioral predictors of injurious pecking: exploration of behavioral profiles in two commercial strains of laying hens

**DOI:** 10.1016/j.psj.2026.107998

**Published:** 2026-10-20

**Authors:** Lucille Dumontier, Vitor H.B. Ferreira, Gaëlle Lefort, Auriane Foreau, Julie Lemarchand, Fabien Cornilleau, Maxime Quentin, Vanessa Guesdon, Maryse Guinebretière, Aude Kleiber, Ludovic Calandreau

**Affiliations:** a INRAE, CNRS, IFCE, Université de Tours, Centre Val de Loire UMR Physiologie de la Reproduction et des Comportements, Nouzilly, 37380, France; b Technical Institute for Poultry (ITAVI), Nouzilly, 37380, France; c JUNIA, Comportement Animal et Systèmes d’Elevage, Lille, France; d Epidemiology, Health and Welfare Unit, French Agency for Food, Environmental and Occupational Health & Safety (ANSES), Ploufragan, 22440, France

**Keywords:** Injurious pecking, Laying hen, Early behavior, Behavioral profile, Longitudinal study

## Abstract

Injurious pecking remains one of the most persistent welfare and economic challenge in the egg industry. Despite decades of research, its multifactorial nature makes it hard to prevent and manage. In this longitudinal experimental study, we used an innovative approach to identify the behavioral profile of high peckers (P+) compared to low peckers (P-) in two strains of Lohmann laying hens: the Lohmann LSL classic and the Lohmann Brown classic (N = 90 per strain). These animals were tested in two test rounds at 5-10 weeks and 18-23 weeks of age to measure a panel of behavioral traits. Each round consisted of the following tests: detour, novel object, cognitive bias, multivariate and tonic immobility, aiming to characterize different aspects of birds’ behavioral profile. Home pen behavioral observations were also performed to establish the time budget of the individuals. Between 31-38 weeks of age, pecking observations were performed to identify P+ and P- birds (6.5 hours per individual). Data were analyzed using (sparse) partial least square discriminant analysis ((s)PLS-DA) to identify variables that best characterized our two strains, and which best discriminate P+ from P- birds for each strain (20 birds pecking the most and the least were retained for the analysis). Results show that, overall, LSL birds were more active than Brown birds, which exhibited a more passive behavioral profile. For Brown birds, the model was able to classify birds as P+ or P− with an error rate of 26% and 35%, respectively. When considering the 18 most relevant variables, P+ birds appeared less fearful and more socially motivated than P-. For LSL birds, the model was able to classify birds as P+ or P- with an error rate of 30% and 48%, respectively, suggesting that these results should be interpreted with caution. These results highlight the complexity of predicting feather pecking and the importance of taking the strain into account when trying to identify reliable indicators.

## Introduction

Despite decades of research, injurious pecking (IP) remains a major challenge for the egg industry. This damaging behavior involves forceful pecks directed at the head, neck, feathers or skin of a conspecific (Savory, 1995; Widowski and Rentsch, 2022). It frequently results in plumage damage, tissue injury, pain, cannibalism and, eventually, death (Green et al., 2000). Beyond its negative impact on animal welfare, IP can also cause economical losses to the producers by reducing egg production (Huber-Eicher and Sebö, 2001), increasing the mortality within the flock (Blokhuis et al., 2007) and altering birds’ thermoregulation ability, resulting in an increase in feed intake to compensate for the lack of feather coverage (Peguri and Coon, 1993).

IP is considered as a multifactorial behavior arising from complex interactions between genetic predisposition, early-life and current environment, social dynamics and neurobiological regulation, making it difficult to prevent and manage (Cronin and Glatz, 2021). Current detection methods, such as assessing plumage or body condition, are often implemented too late to mitigate pecking outbreaks. While beak trimming, a practice consisting in treating the beak of the animals to remove the sharp tip, can help reduce IP and its consequences, it does not fully prevent it. Indeed, a study led on French flocks showed that one-third of the birds observed had plumage damage despite being beak trimmed, and that measures taken during outbreaks failed to completely contain IP (Mika et al., 2020). In addition, because of its negative consequences on animal welfare (see the report by EFSA Panel on Animal Health and Animal Welfare (2023)) and increasing civil society concerns, beak trimming is already banned, or is currently being phased out in several European countries, and the egg industry is transitioning towards cage-free systems, bringing new challenges in terms of flock management. Therefore, early detection methods are crucial for implementing preventive measures and ensure good welfare of the flocks.

A promising lead would be the identification of early behavioral predictors of IP. Indeed, studies led on experimental lines selected on their propensity to peck showed several behavioral differences between lines, with individuals from high pecking lines (HFP) being more proactive, *i.e.* more exploratory, bolder and less flexible, than individuals from low pecking lines (LFP) (Korte et al., 1997, 1998). For example, there is a positive relationship between locomotory behaviors and IP in these lines, with high peckers walking more in a novel environment than low peckers (Kjaer, 2009). Individuals from high pecking lines also showed more resistance (*i.e.*, shorter durations) in a tonic immobility test, and had a faster heart rate and stronger physiological responses than the ones from low pecking lines (Korte et al., 1997, 1998; van der Eijk et al., 2018). This proactivity is also shown in other contexts, such as in the social isolation test, during which birds from high pecking lines vocalized more (Rodenburg and Koene, 2003), indicating a stronger motivation to reinstate social contact (Forkman et al., 2007). Cognition-wise, a study showed that HFP birds were slower to associate a colored bowl with a food reward than LFP birds, and had poorer inhibitory control in a Go/No-Go task (Pichová et al., 2021). Given that certain behavioral traits seem to be associated with IP, some individuals could show a predisposition to become peckers. By identifying these traits, it could be possible to identify them in young individuals, before they start performing IP.

However, to the best of our knowledge, little information is available at the individual scale in commercial strains used by the egg industry. In studies investigating this topic, it has been shown that fearful pullets were more at risk of becoming peckers during the production phase (Rodenburg et al., 2013). These results are thus contradictory to what is observed in HFP lines, which are described as less fearful and more proactive. This suggests that there is a complex relationship between temperament and IP, and differences at the individual level could be a key to better understand and manage this behavior.

In this study, we aimed at identifying early behavioral traits associated with the later expression of injurious pecking in two strains of laying hens housed separately, the Lohmann LSL classic and the Lohmann Brown classic, as plumage coloration seems to affect the birds predisposition to perform IP (de Haas et al., 2014a). We first tested the birds in two test rounds to collect behavioral measures and performed home pen observations. We then characterized the behavioral profile of both strains, and identified early behavioral traits associated with later IP in each of these two strains. We hypothesized that the two strains will exhibit different behavioral profiles, and that high peckers (P+) would differ from low peckers (P-) in specific behavioral traits. As brown hens are known to perform more IP than white hens, we expect to observe more IP in Brown than LSL birds, and to identify P+ more easily and reliably in Brown than in LSL birds.

## Materials and methods

### Ethical note

This study was conducted at the Pôle d’Expérimentation Avicole de Tours (UE PEAT, INRAE, Experimental Poultry Facility, https://doi.org/10.15454/1.5572326250887292E12) at the INRAE Val-de-Loire Center, France, from January to October 2024. The study received approval from the Direction Générale de la recherche et de l'innovation under project number APAFIS #44361-202308031059962, in compliance with current French legislation.

### Animals and housing

Female chicks (*Gallus gallus domesticus*) of Lohmann LSL Classic (N = 177; white feathered) and Lohmann Brown Classic (N = 173; brown feathered) were received at our Experimental Poultry Facility at one day of age. They were housed in the same building in two adjacent brooder rooms measuring 4 × 4 m with an initial stocking density of 11 chicks/m². The floor was covered with wood shavings, and feed and water were provided *ad libitum* through three round feeders (Ø 40 cm) and a water line with automatic drinking nipples. During the first few days, feed was scattered on chick paper and water was provided via additional bell drinkers to facilitate their access. At four days of age, each bird was individually identified with a lightweight wing-tag attached to the wing membrane. At eight days of age, two elevated cardboard platforms (58.5 × 38.5 cm) and a perch (100 cm length) were introduced as enrichments. The light and heating program started with continuous light and high temperatures (34°C) on the first day, and were gradually reduced over days, reaching 12 hours and 26°C by 28 days of age.

At four weeks of age, 200 chicks (100 LSL and 100 Brown) were randomly selected and transferred to another building (∼200 m from the brooder), where they remained until the end of the experiment.

They were all floor-housed in the same rearing room and divided in same-strain social groups of ten individuals. The strains were distributed in the room so that pens housing Brown birds were always next to pens housing LSL birds (in total, 10 pens per strain). Each pen measured 170 cm x 160 cm (3.7 birds/m²) and was furnished with a perch (100 cm length) and three nests (33 cm x 43 cm x 40 cm, length x width x height). At 12 weeks of age, an additional perch was added to each pen (total perch length: 200 cm). Wood shavings were used as litter, and pens were separated by opaque screens to minimize visual contact between pens. Feed and water were provided *ad libitum* through a round feeder (Ø 40 cm) and an automatic drinking nipple water line. The day following their transfer to the new building, all individuals were equipped with a lightweight numbered backpack matching their feather color to facilitate their identification. To accommodate growth, the backpacks were replaced with a larger size at 17 weeks of age. Backpacks were regularly checked, adjusted and replaced when necessary to ensure they did not cause any discomfort to the birds.

The heights of the water line and feeder were regularly adjusted to match chick growth, and feed composition was adapted to meet the nutritional requirements of each developmental stage (Sanders Ouest, Site de Champagné, France). Light and temperature settings were gradually adjusted to reach an average temperature of 20 °C by six weeks of age, while light exposure gradually increased to prepare the birds for egg-laying, ending with a 16:8 light:dark cycle. To monitor their growth, birds were weighed regularly throughout the study period: at 4 days of age and 4, 10, 14, 17, 23, 27, 30, 33 and 40 weeks of age. At the end of the experiments, birds were rehomed.

### Experimental design

For each strain, the birds from nine pens (N = 90 birds per strain) were tested in two identical rounds of tests to assess their behavioral profile, completed by behavioral observations in their home pens (Fig. 1). The birds from the remaining pens were used as social stimuli during the tests requiring social motivation. The first test round ran from 5 to 10 weeks of age and the second test round from 18 to 23 weeks of age. From 31 to 38 weeks of age, observations were performed to record pecking behavior. The behavior in their home pens were observed on 10 occasions, between 5 and 23 weeks of age.

**Fig. 1:**
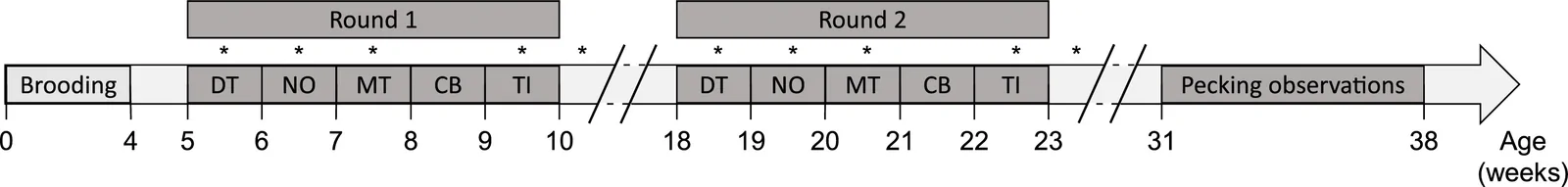
Experimental design timeline showing the two test rounds and the pecking observations. The asterisks (*) represent the behavioral observations performed in the home pens once a week. *DT: Detour Test; NO: Novel Object test; MT: Multivariate Test; CB: Cognitive Bias test; TI: Tonic Immobility test.*

During each round of test, five tests assessing various behavioral traits were performed: a detour test, a novel object test, a multivariate test, a cognitive bias test and a tonic immobility test (see details in the respective subsections below). For all tests performed in a testing arena, the birds were gently captured in their pens and placed individually in an opaque transport box (35×25×31 cm) to be brought to the testing room, located roughly 10 m away from the rearing room, within the same building. After testing, they were returned to their pen in the same transport box and the arena was thoroughly cleaned. The testing room contained two testing arenas, allowing to test two birds simultaneously with no visual contact (one Brown and one LSL). All tests were recorded via a camera (GoPro HERO 11 or 12) to be later analyzed. Birds were always tested in a predetermined pseudo-randomized order to maintain a balanced testing frequency across pens throughout the day. All video analyses and behavioral observations were performed by the same experimenter.

### Behavioral and cognitive tests

***Detour Test (5 & 18 weeks of age).*** The detour test, usually used to measure spatial ability and behavioral inhibition (Norman et al., 2019), was performed over two testing days. At the beginning of the test, the bird was placed in a U-shaped compartment with a grid front (Round 1: 34×25 cm; Round 2: 40×40 cm), facing a mirror (Round 1: 30×30 cm; Round 2: 60×60 cm) and a Petri dish containing three live mealworms (Saint Laurent, La Chapelle-Saint-Laurent, France). These two acted as social (mirror) and food (mealworms) motivations. To take into account birds’ growth, it was conducted in a small cardboard arena (120×120 m, Fig. 2A) during the first round of test and a larger one (250×300 cm, Fig. 2B) during the second round of test. The latency for the bird to make the detour, *i.e.*, to leave the compartment and cross its front (thin dotted line on Fig. 2), was recorded with a maximum trial duration of five minutes. In case the bird did not perform the detour, it was attributed the maximum latency of 300 s.

**Fig. 2:**
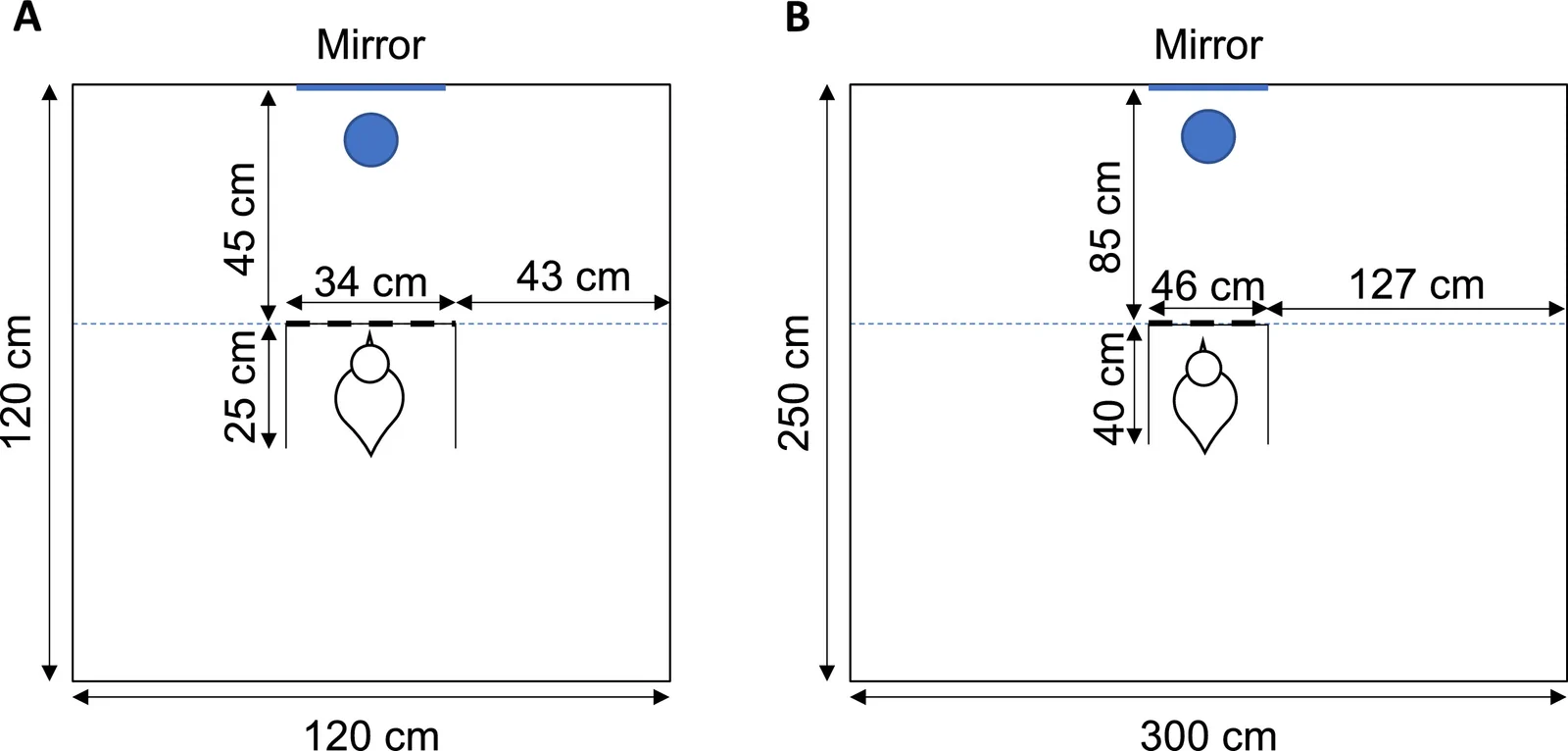
Schematic representation of the detour arena used during the first round of test (A) and the second round of test (B). The blue circles represent the Petri dish containing the mealworms used as food motivation.

***Novel Object Test (6 & 19 weeks of age).*** The novel object test, traditionally used to measure fear (Forkman et al., 2007), and more particularly neophobia, was performed directly in the home pen of the birds on a single morning. An object the birds had never encountered before was introduced in their home pen (Round 1: small clay pot; Round 2: PVC tube with colorful stripes). A virtual zone close to the novel object (within 55 cm) was defined (Fig. 3), and scans were performed every 10 seconds for two minutes to record the position of the birds. For each bird, the proportion of scans in the zone close to the novel object was used for subsequent analyses.

**Fig. 3:**
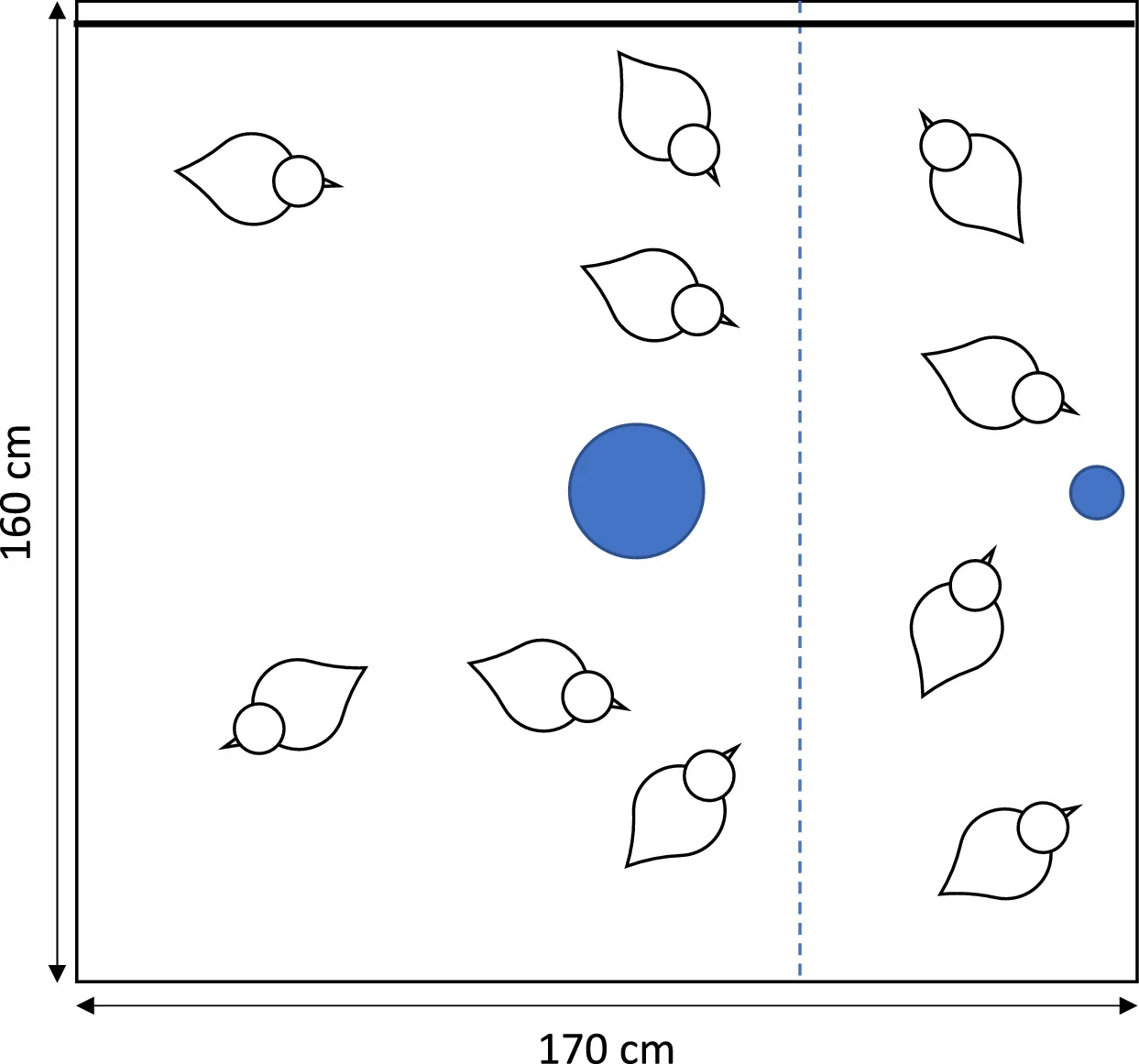
Schematic representation of the novel object test performed in the home pen. The largest circle represents the feeder, and the thick black line the water line. The smaller circle represents the novel object (Round 1: small clay pot; Round 2: PVC tube with colorful stripes). The dotted lines indicate the virtual zone of proximity with regards to the novel object.

***Multivariate Test (7 & 20 weeks of age).*** The multivariate test was performed in a 250×300 cm arena and allowed to measure social motivation, exploration, and foraging (Bonnefous et al., 2023; Zidar et al., 2018). The test took place over two days and one afternoon during the first test round and two days during the second round of tests. The arena contained a central compartment (Ø 50 cm) containing two stimulus hens (replaced every two hours), and four cardboard panels facing each other two by two (Fig. 4). This allowed to delimitate three zones in the arena: “central zone”, close to the conspecifics, the “outer zone”, further away from the conspecific but the tested bird could maintain visual contact, and the “behind a screen” zone, where the bird lost visual contact with its conspecifics. The floor of the arena was covered with wood shavings, encouraging foraging and exploration. The tested bird was placed between two panels, facing the middle of the arena, and let to explore for five minutes. The time spent in each zone and the time spent foraging were recorded, alongside with the number of transitions between the zones. The bird was considered in one of the zones when its center point entered the zone. Due to some stimuli birds escaping from their compartment during the second round of tests, three birds were retested the day following the last day of testing (all LSL birds). Due to technical issue, the trial of one Brown bird only lasted four minutes.

**Fig. 4:**
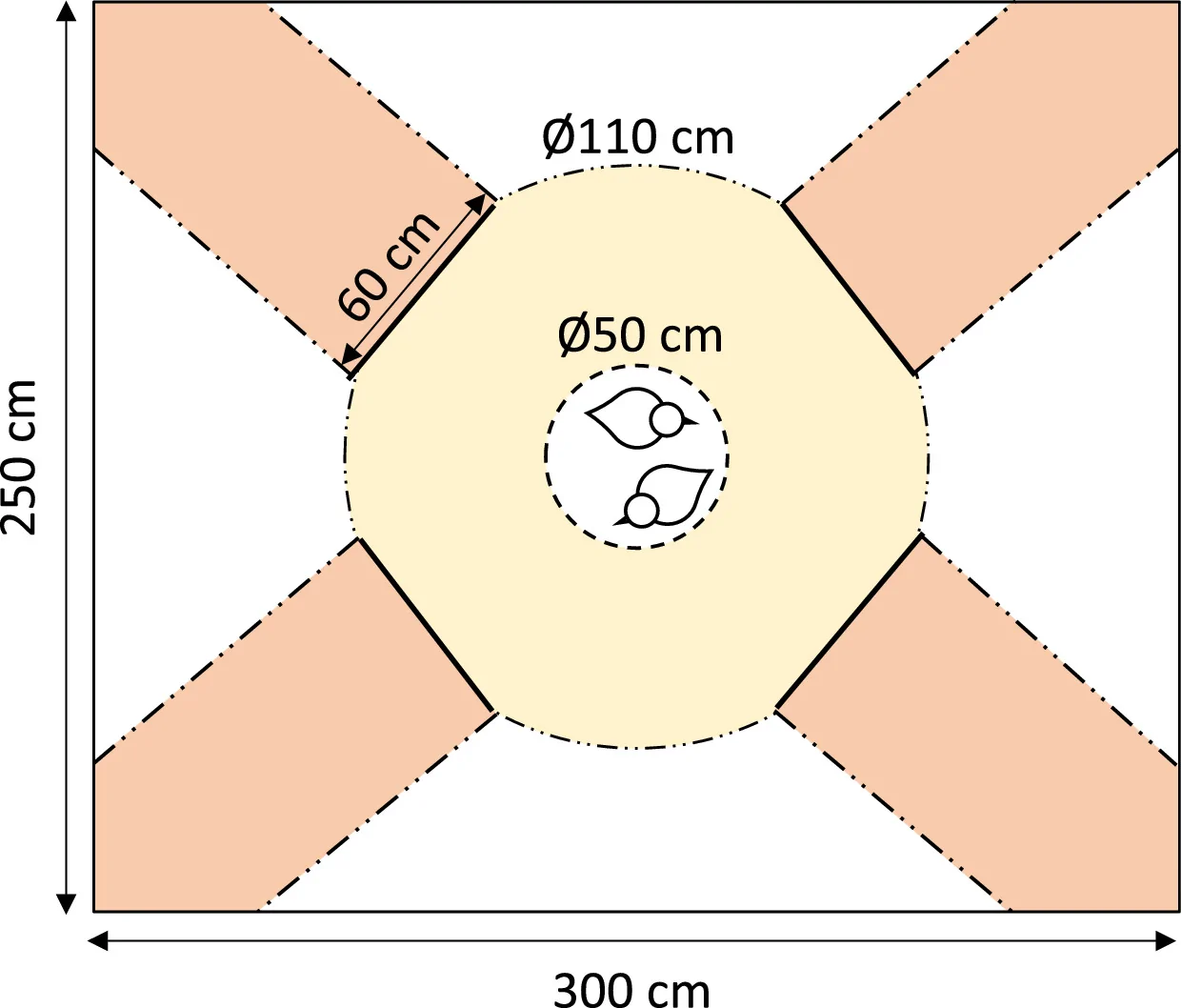
Schematic representation of the multivariate test arena used during the two rounds of tests, showing the conspecific compartment, the “inner” zone (in yellow), which allowed visual contact while being in close proximity to the conspecifics, the “outer” zones (in white), which still allowed visual contact with the conspecifics while being further away, and the “behind a screen” zones (in orange), where no visual contact with the conspecifics was possible.

***Cognitive Bias Test (8 & 21 weeks of age).*** The cognitive bias test is commonly used to determine how individuals perceive their environment and whether they are rather pessimistic or optimistic. In this modified version based on Hedlund et al. (2022) and Ferreira et al. (2024), we used four different stimuli: a mirror, a picture of a conspecific at the same age (positive), a picture of an owl (negative) and a morph image of the owl picture and the conspecific picture (ambiguous). The latter was crafted with the FotoMorph software. The arena consisted of a corridor-like apparatus in which the bird was released at one end and exposed to the different stimuli at the other end (Fig. 5). Birds were first exposed to the mirror, which served as a social motivator, enabling them to familiarize themselves with the arena and learn that reaching the end of the corridor granted access to two conspecifics and mealworms, while also controlling for potential lack of motivation or locomotor impairments. The conspecifics were placed in a cage (55×100 cm) and replaced between each stimulus change, so that they never stayed in the testing arena for more than two hours. After that, the birds were exposed to the conspecific picture, and then the owl picture (or vice versa, exposition order balanced across strain and day), and finally the morph picture. The birds were exposed to all the stimuli on the same day and returned to their home pen between each trial (∼2 hours between trials). The test took place over four days during the first test round (44-46 birds per day) and five days during the second round of tests (36 birds per day). The latency to reach the end of the corridor was recorded for each trial, and the birds were attributed the maximum latency of 180 s if they did not reach the end of the corridor withing three minutes. All images were printed in high quality in color against a white background. To reinforce the frightening effect of the stimuli, the size of the owl pictures was increased compared to the conspecific picture, with the morph picture having an intermediate size.

**Fig. 5:**
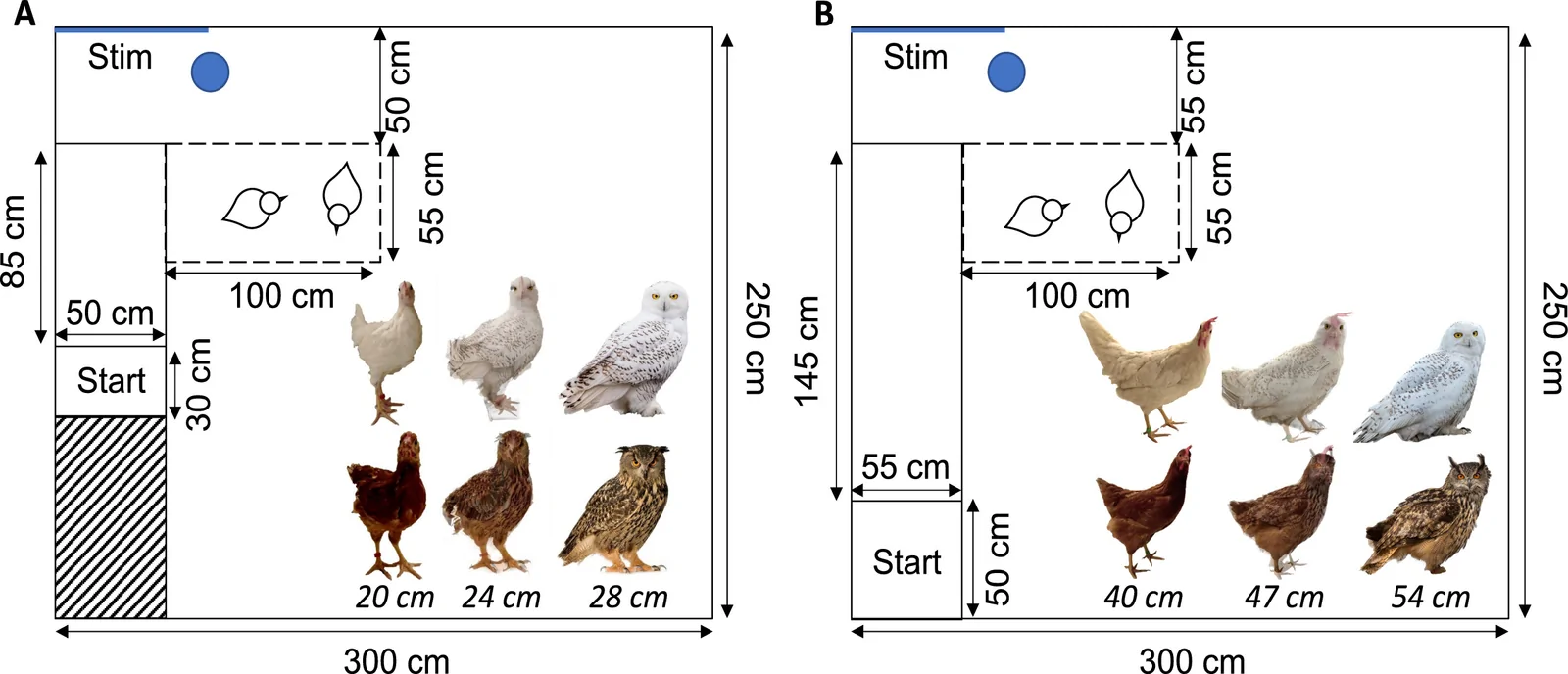
Schema of the cognitive bias arenas used during the first round of test (A) and the second round of test (B), showing the different stimuli used for each strain with their respective height. The stimuli sizes were adjusted between the two test sessions to accommodate birds’ growth. The blue circles represent the Petri dish containing the mealworms, and “Stim” stands for stimulus position.

***Tonic Immobility Test (9 & 22 weeks of age).*** The tonic immobility test is a standard measure of fear and proactivity in chickens (Forkman et al., 2007). It took place over three mornings (30 birds per day) in the testing room, which provided auditory isolation from the conspecifics. As tonic immobility responses are very sensitive to variations in handling, it was always induced by the same experimenter. The bird was taken out of the transportation box and placed on its back in a cradle covered by a cloth, while the experimenter applied a firm pressure on its breast. After 10 seconds, the experimenter slowly removed her hands from the bird. If the bird rightened itself within 10 seconds, the tonic immobility was considered not successfully induced and the procedure was repeated, with a maximum of three inductions. Otherwise, the test ran for five minutes, or until the bird rightened itself, whichever occurred first. The number of inductions and the tonic immobility duration (i.e., the latency to rightened after a successful induction) were recorded for each bird. The birds were attributed the shortest duration of tonic immobility (0 s) if it was not successfully induced after the third induction, and the maximum duration (300 s) if it was still in tonic immobility at the end of the test.

### Behavioral observations in the home pen

Behavioral observations were performed once a week, at 5, 6, 7, 9, 10, 18, 19, 20, 22 and 23 weeks of age (Fig. 1), leading to a total of five observation sessions per round of test. During the observation, the experimenter sat motionless in a chair by the pen door, allowing her to see the full pen without moving. After a three minutes habituation period to the experimenter, each observation session lasted for 15 minutes. Scans were performed every 3 minutes to record the behavior of each individual in the pen (see ethogram in Table 1, adapted from Paul, et al. (2022)). The observation order was semi randomized over the observation days so that the observation sessions were overall balanced between morning and afternoon.

**Table 1:** Ethogram used during the behavioral observations. Adapted from Paul et al. (2022).

Category	Behavior	Description
Feeding	Eating	Pecking and/or eating at feeder.
	Drinking	Pecking and/or drinking at drinker.
Foraging	Foraging	Pecking and scratching in litter.
Inactive	Resting	Sitting or standing inactive with (partially) closed eyes or head tucked under wing.
	Perching	Standing/sitting on the perch.
	Sitting	Sitting with open eyes.
	Standing inactive	Standing inactive with open eyes, relaxed posture, head close to the body.
Alert	Standing alert	Standing inactive with open eyes, upright neck and body.
Locomotion	Walking/running	Bird takes more than one step forward.
Comfort behavior	Preening	Bird arranges or oils her feathers with her beak Raise feathers and clean and realign them with beak.
	Wing/Leg stretching	Bird stretches its wing/leg.
	Dustbathing	Lie on side, scratch at pen floor, rub head and neck on floor, open wings.
	Flap wings	Movements of the wings on both sides.
Nest	Nesting	Bird is out of sight in the nest box.

For each test round, the occurrence ratio of each behavior type was calculated as the number of scans in which the behavior was observed divided by the total number of scans performed during the behavioral observations (e.g., 5 scans spent foraging out of 30 total scans).

### Moderate stressor and scoring of pecking behavior

Starting from 31 weeks of age, pecking observations were performed (Fig. 6). As for the behavioral observations, the experimenter sat motionless by the door of the pen for 15 minutes, after a three-minutes habituation period. All interactions between birds involving IP were recorded during the 15-minutes period, with the IDs of the pecker. IP was defined as forceful pecks directed towards any body parts of the another individual. Grabbing and pulling of feathers can occur, with sometimes ingestion. The recipient often reacts to the pecks (vocalization, escape). The observations were done twice a week at 31, 32 and 33 weeks of age to assess the amount of pecking in the pens. Then, due to low levels of severe feather pecking observed, daily observations were performed as we manipulated the light intensity at 34 and 35 weeks of age. The light intensity was first increased from 20 to 50 lux for three days, then to 100 lux for seven days. It was then lowered to 20 lux for 11 days, and daily observations were performed for 10 days as we increased the light intensity to 100 lux at 37 and 38 weeks of age. The purpose was to mimic what could occur in a laying farm and induce a moderate stress to the birds, potentially inducing more injurious pecking.

**Fig. 6:**
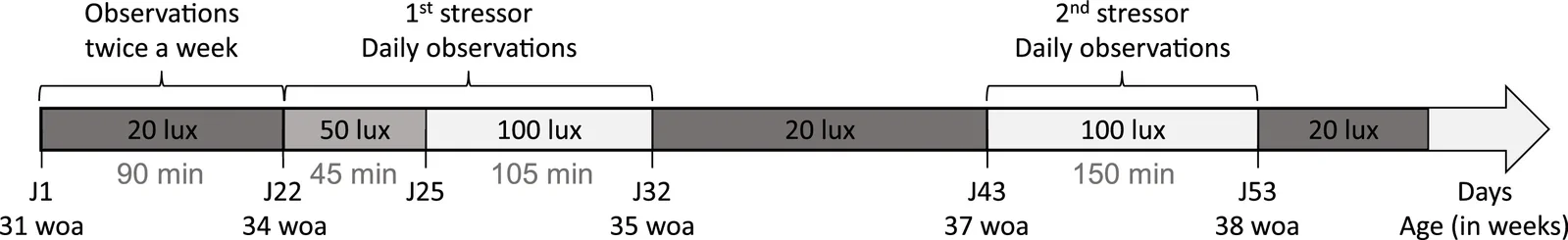
Timeline showing the light treatment applied to the birds from 31 weeks of age (woa) and the behavioral observations performed at each stage, with the amount of observation time in minutes per bird.

In total, each bird totalized 405 minutes of observation. The sum of all injurious pecks given by each bird was calculated at the end of the observations.

### Statistics

Statistical analyses were conducted using R version 4.5.2 (R Core Team, 2025).

Because some birds were transferred to a recovery pen and later rehomed due to injuries over the course of the experiment, individuals with less than four hours of recorded pecking behavior were excluded from the analysis, as this amount of data was deemed insufficient to reliably classify birds as peckers or non-peckers. This led to a final sample size of 82 LSL and 85 Brown birds.

All the variables from the two rounds of tests were merged in a single dataset consisting of one row per individual, and one column per variable. We first ran a correlation matrix on our dataset to identify highly correlated variables (functions cor() and flattenCorrMatrix()). Variables with a correlation coefficient higher than 0.8 or lower than −0.8 were removed from the dataset (see the relevant section below). A table listing all variables and their names is available in the supplementary material (Supp. 1).

Then, a partial least squares discriminant analysis (PLS-DA) was applied to our dataset to identify behavioral variables characterizing our two strains of hens. This supervised multivariate analysis method computes latent components that maximize covariance between the predictor variables (behavioral measures) and a categorical response variable (in this case, the strain), encoded as dummy variables. After assessing the model performances using cross-validation when increasing the number of components, we set the number of components to the one with the lowest error rate. The final PLS-DA model had two components and we used 10-folds cross-validation with 100 repeats to assess the classification performance.

Finally, sparse PLS-DAs (sPLS-DA) were applied to our data (one analysis per strain) to identify variables which best discriminate high peckers from low peckers. This variant of the PLS-DA constraints the model so that only a subset of variables with the strongest discriminatory power is retained. For each strain, we selected the 20 birds which pecked the most (P+) and 20 birds which pecked the least (P-) to perform our analyses on the extreme and get clearer behavioral profiles (see Fig. 7 for the distribution of pecking). As we decided to retain all birds giving the same number of pecks within each category, this led to a sample size of 37 LSL birds (20 P+, at least 8 pecks given; 17 P-, 0 pecks given) and 41 Brown birds (20 P+, at least 17 pecks given; 21 P-, less than 4 pecks given). The tune.splsda() function was used to tune the analysis and select the optimal number of components and variables. To prevent the sPLS-DA to run into singularity issues for the LSL birds, the maximum number of variables to include in the analysis was set to 36 in place of the 37 total variables. The choice of optimal values for the sparsity parameters and the evaluation of classification was performed using a 6-fold cross-validation and 100 repeats.

**Fig. 7:**
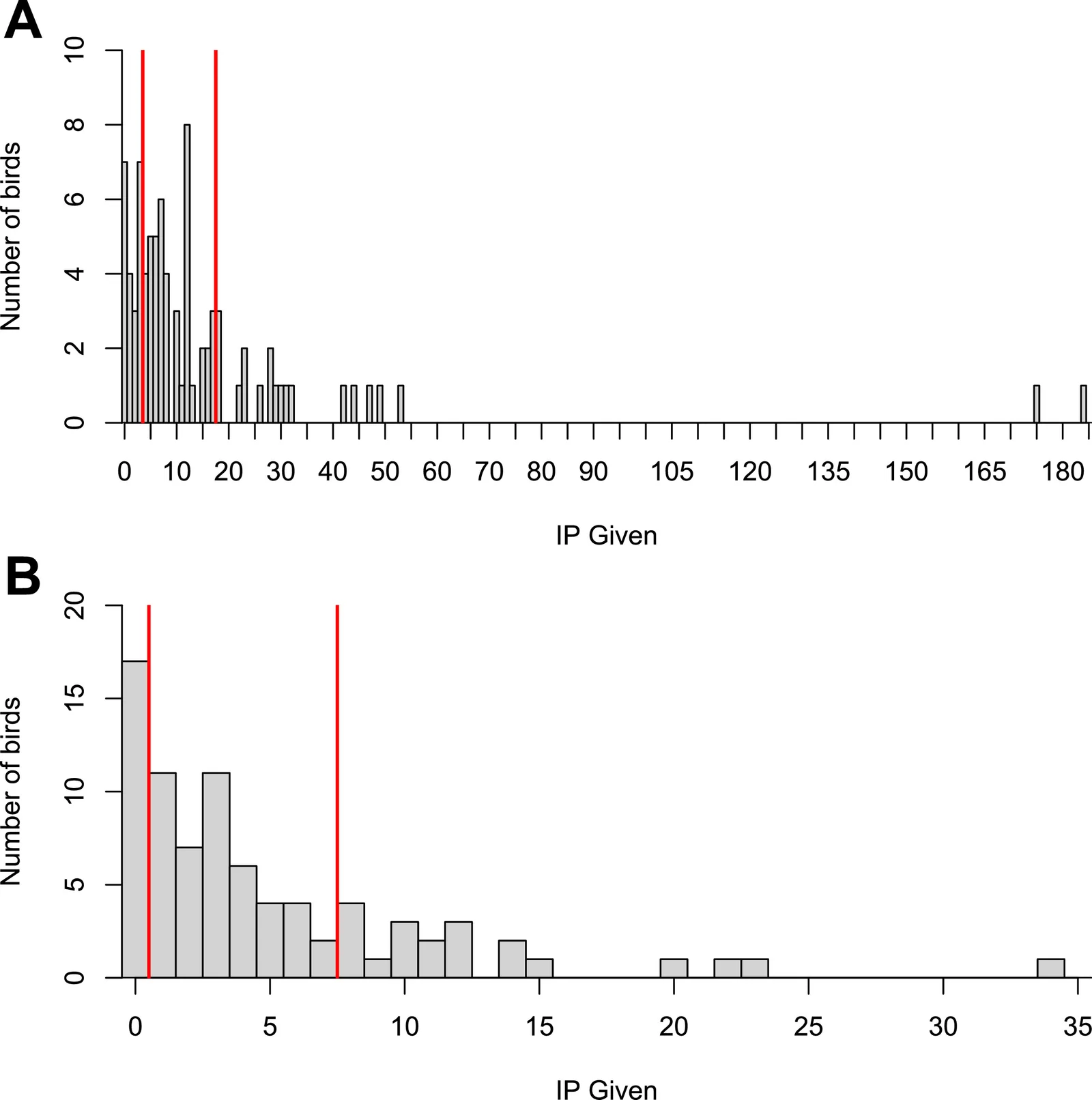
Distribution of the number of injurious pecks (IP) given by (**A**) Brown and (**B**) LSL birds. Red lines indicate the cutoffs used to define the P- (left) and P+ (right) groups retained for the analyses. Individuals with intermediate values (between the red lines) were excluded.

For each (s)PLS-DA, the variables contributing the most to group discrimination were identified using the vip() function. Variables with a Variable Importance in the Projection (VIP) score greater than 1 were considered the most relevant for characterizing the groups, as specified in the function documentation. For each of these variables, the mean and standard deviation are presented in the text as mean ± sd. The (s)PLS-DA analyses were all performed using the mixOmics R package (Rohart et al., 2017).

## Results

### Correlation between variables

Results from the correlation analysis showed that variables from the multivariate test were highly correlated. Within each test session, the time spent in the inner zone (close to the conspecifics) was correlated with the time spent in the outer zones and behind the screens, as well with the number of transitions between the zones (cor < −0.8). Therefore, only the time spent in the inner zone was kept for further analysis. Only for the second test session, the latency to enter the outer zone and the latency to enter the behind the screen zones were highly correlated (cor > 0.8) and only the former was retained for the subsequent analysis. The correlation matrix is available in the supplementary materials (Supp. 2).

### Behavioral profiles of the strains

Two components were identified to discriminate Brown birds from LSL birds. The first component explained 8% from the variation and the second 7% (Fig. 8A). With these two components, the model was able to classify birds as Brown or LSL with an error rate of 10% and 8.6%, respectively. Overall, 12 different variables were identified as the most relevant (VIP > 1, Fig. 8B & C). It appears LSL birds spent more time foraging during the first multivariate test than Brown birds (78 ± 30 s vs 38 ± 30 s, respectively) and had a higher latency to approach the owl stimulus during the first cognitive bias test (107 ± 82 s vs 57 ± 76 s). LSL birds spent, on average, a lesser proportion of their time budget and a greater amount inside the nest (6 ± 7% vs 3 ± 4%) and expressing comfort behaviors than Brown birds during the first round of tests (12 ± 7% vs 19 ± 10%), while the opposite was observed for the latter during the second test round (18 ± 11% vs 11 ± 8%). However, substantial inter-individual variability was observed in both groups. For both rounds of test, they were overall faster to perform the detour (Round 1: 142 ± 101 s vs 228 ± 97 s; Round 2: 82 ± 81 s vs 193 ± 97 s) and to approach the mirror during the cognitive bias test (Round 1: 3 ± 2 s vs 25 ± 48 s; Round 2: 7 ± 20 s vs 22 ± 42 s). During the second test round, LSL birds spent more time in the zone close to their conspecifics during the multivariate test (248 ± 69 s vs 210 ± 75 s). They also spent a lesser amount of their time budget performing locomotory behaviors (11 ± 7% vs 16 ± 7%) than Brown birds and required, on average, less induction than Brown birds to enter a tonic immobility state (1.35 ± 0.60 vs 1.87 ± 0.84, respectively).

**Fig. 8:**
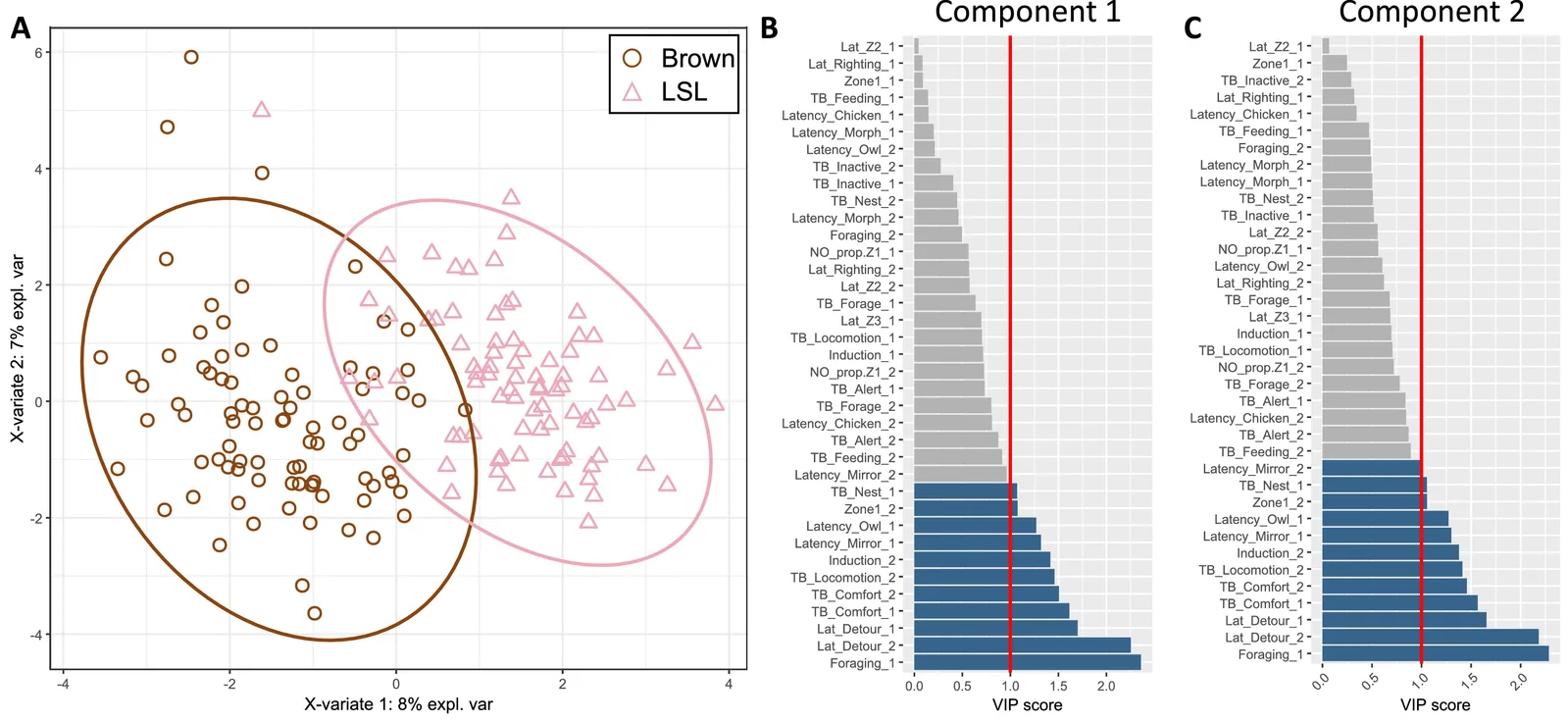
Results from the PLS-DA of the behavioral traits measured during the two test rounds according to the hens’ strain (LSL: N = 82; Brown: N = 85), showing the sample projection of the final PLS-DA (**A**) and the variable importance in projection for component 1 (**B**) and 2 (**C**). Variables with a VIP score greater than 1 are shown in blue.

### Predictors of injurious pecking behavior

***Brown Birds.*** Two components were identified to discriminate P+ from P- birds. The first component explained 11% from the variation and the second 6% (Fig. 9A). With these two components, the model was able to classify birds as P+ or P- with an error rate of 26% and 35%, respectively. Overall, 18 different variables were identified as the most relevant (VIP > 1, Fig. 9B & C). During the first test round, P+ spent a similar amount of their time budget being inactive (32 ± 11% vs 30 ± 12%) and alert (5 ± 4% vs 3 ± 3%), and they spent a lesser amount of their time budget performing feeding behaviors (14 ± 8% vs 21 ± 10%) than P- birds. Compared to P- birds, P+ birds tended to spend a greater amount of their time budget performing comfort behavior (Round 1: 21 ± 9% vs 16 ± 8%; Round 2: 15 ± 11% vs 10 ± 6%) and a lesser amount foraging (Round 1: 13 ± 7% vs 17 ± 8%; Round 2: 21 ± 11% vs 27 ± 12%). Responses to the conspecific picture during the cognitive bias test were consistent across rounds, with P+ birds taking on average more time to approach the stimulus than P- birds (Round 1: 46 ± 63 s vs 19 ± 42 s; Round 2: 102 ± 87 s vs 48 ± 72 s). P+ birds were slower to approach the ambiguous stimulus (morph picture) during the first test round than P- birds (32 ± 57 s vs 16 ± 38 s), but faster during the second round (32 ± 57 s vs 44 ± 68 s), with P- increasing their latency to approach the stimulus. For both test rounds, P+ birds had an overall shorter tonic immobility duration than P- birds (Round 1: 118 ± 89 s vs 161 ± 98 s; Round 2: 100 ± 101 s vs 175 ± 133 s). For the second test round only, P+ birds had a higher latency to approach the mirror during the cognitive bias test (38 ± 62 s vs 11 ± 11 s) and spent a greater amount of their time budget performing locomotory behaviors (20 ± 8% vs 13 ± 7%). Finally, during the second multivariate test, P+ spent more time in the zone close to their conspecifics (222 ± 73 s vs 172 ± 78 s), had a higher latency to enter the zone further away (166 ± 110 s vs 114 ± 97 s) and spent less time foraging (47 ± 32 s vs 74 ± 60 s), although inter-individual variability was observed.

**Fig. 9:**
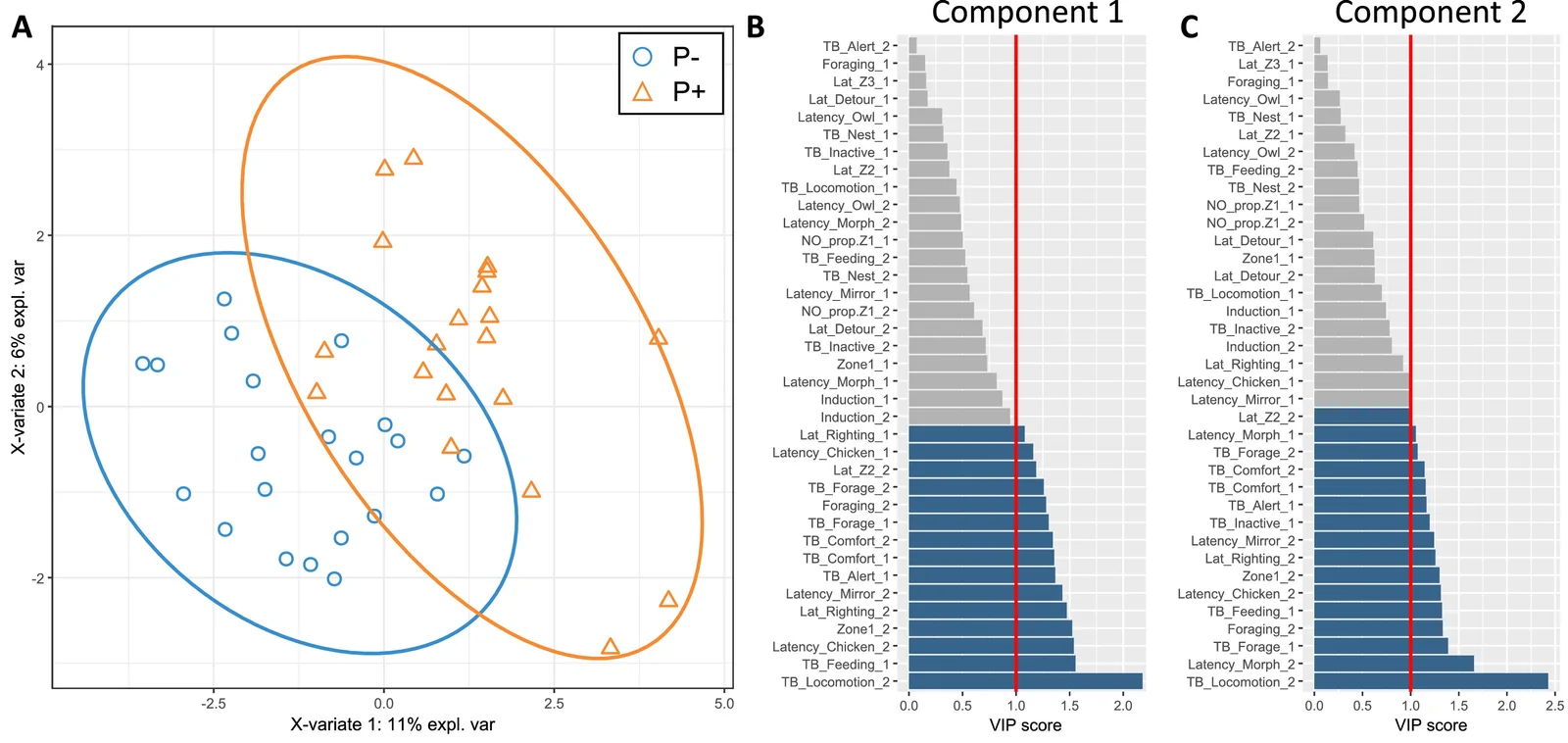
Results from the sPLS-DA of the behavioral traits measured for the Brown birds during the two test rounds according to their pecking group (P+: N = 20; P-: N = 21), showing the sample projection of the final sPLS-DA (**A**) and the variable importance in projection for component 1 (**B**) and 2 (**C**). Variables with a VIP score greater than 1 are shown in blue.

***LSL Birds.*** One component was identified to discriminate P+ from P- birds, which explained 7% of the variation (Fig. 10A). With this component, the model was able to classify birds as P+ or P- with an error rate of 30% and 48%, respectively. Overall, 13 different variables were identified as the most relevant (VIP > 1, Fig. 10B). During the first test round, P+ birds spent on average a lesser amount of their time budget being inactive (25 ± 12% vs 33 ± 13%) and a greater amount feeding (19 ± 9% vs 14 ± 16%) and performing locomotory behaviors (14 ± 8% vs 10 ± 6%) than P- birds. During the first multivariate test, P+ birds had a shorter latency to enter the zone further away from the conspecifics (221 ± 91 s vs 255 ± 70 s) and the zones behind the screens (292 ± 27 s vs 300 ± 0 s) than P- birds, although inter-individual variability was observed. They were also faster, during the first test round, to perform the detour (109 ± 79 s vs 179 ± 106 s) and spent a greater amount of the scans in the zone close to the novel object (88 ± 14% vs 82 ± 16%). For the cognitive bias test, P+ birds approach the chicken stimulus faster than P- birds during the first round of test (17 ± 26 s vs 46 ± 66 s) and slower during the second test round (121 ± 78 s vs 76 ± 81 s). During the second test round, they spent, on average, a lesser amount of their time budget being alert (3 ± 3% vs 5 ± 4%) and spent more time foraging during the multivariate test (71 ± 34 s vs 58 ± 27 s). Finally, P+ birds required more inductions to enter a tonic immobility state (1.25 ± 0.44 vs 1.06 ± 0.24) and had an overall shorter duration of tonic immobility (148 ± 100 s vs 196 ± 109 s) during the second round of tests.

**Fig. 10:**
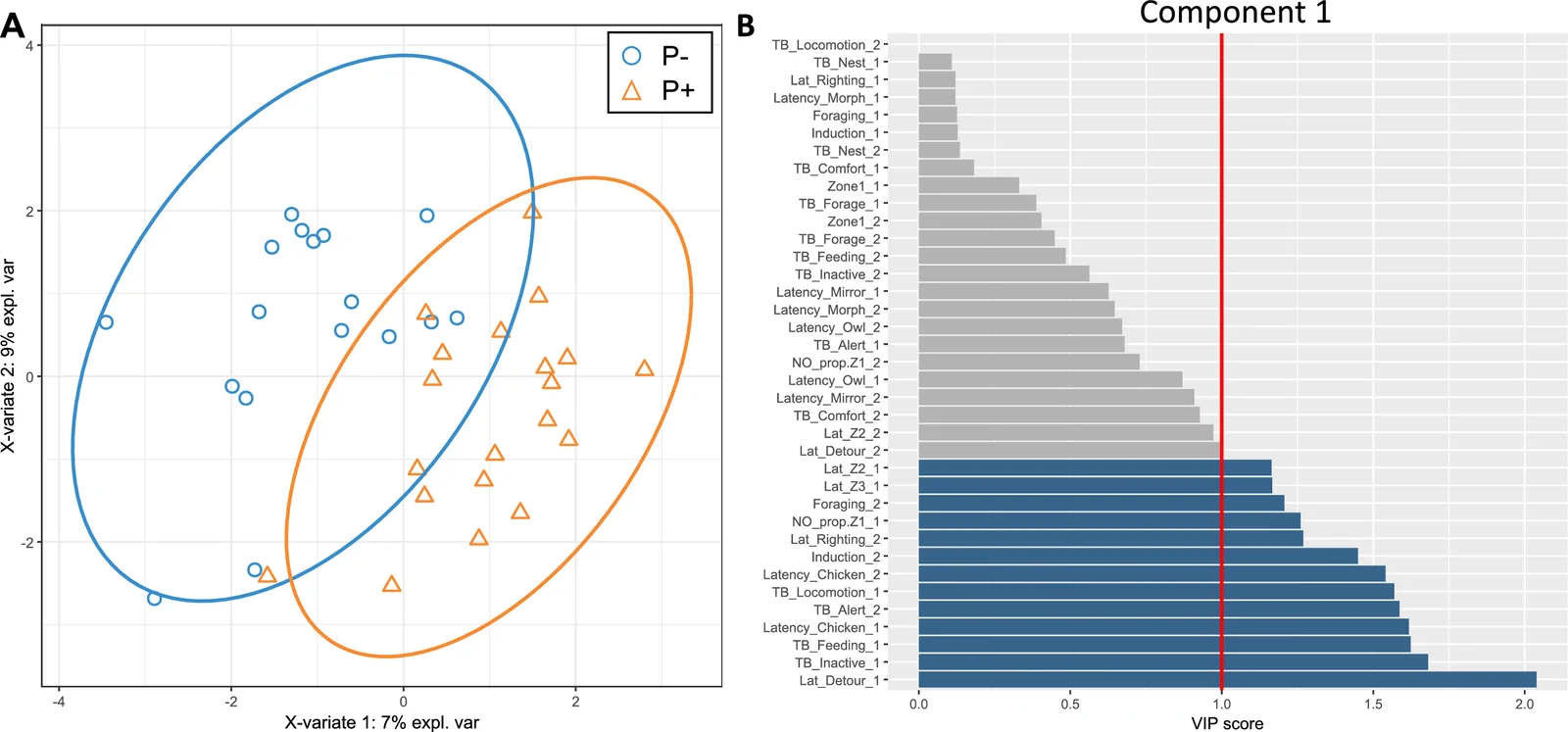
Results from the sPLS-DA of the behavioral traits measured for the LSL birds during the two test rounds according to their pecking group (P+: N = 20; P-: N = 17), showing the sample projection of the final sPLS-DA (**A**) and the variable importance in projection for component 1 (**B**). Variables with a VIP score greater than 1 are shown in blue. *Note that the component 2 is only included for data visualization on the sample projection.*

## Discussion

The aim of our study was to identify early-behavioral profiles of future high peckers (P+) compared to low peckers (P-) in two different strains of laying hens: the Lohmann LSL classic and the Lohmann Brown classic. We first characterized these two strains, and the results from the PLS-DA showed that, as expected, Brown and LSL birds had distinct behavioral profile, which allowed the model to classify the birds by strain with an error rate of 10%. **LSL birds** appeared to be more active than Brown birds, as shown by the shorter latencies to perform the detour and to approach the mirror, as well as by the higher proportion of time spent foraging during the first multivariate test. However, activity alone might not be the only factor affecting the latencies to perform the detour, as LSL birds had a higher success rate to the task than Brown birds (see Supp. 3 for the percentages of success). LSL birds also appeared as more fearful, as they required fewer inductions to reach a TI state during the second round of test and spent more time in the zone close to the conspecifics during the second multivariate test. The higher latency to approach the owl picture during the first cognitive bias test can also suggest higher fearfulness, since this stimulus has been shown to be aversive to the birds (Hedlund et al., 2022). These results are consistent with existing literature, where white breeds are often considered flightier than brown breeds (Jones, 1995; Manet et al., 2023). However, selected variables related to the time budget in the home pen do not necessarily support these findings. Indeed, LSL birds spent a lesser amount of their time budget walking during the second test round than Brown birds and spent more time in the nest during the first test round, suggesting a less active profile. Taken together, these results might suggest that LSL birds have a stronger response to social isolation, which is reflected by higher activity and motivation to reinstate social contact during the tests, but not necessarily observed in the home pen behavior.

These differences in behavioral profile led us to perform a separate sPLS-DA for each strain, aiming to identify the most discriminative behavioral profile between P+ and P- birds within each strain. The model successfully classified Brown birds as P+ or P- with error rates of 26% and 35%, respectively. However, classification performance was much lower for LSL birds, with error rates of 30% for P+ and 48% for P- birds. This discrepancy in model performance may be explained by the fact that brown-feathered birds are generally reported to perform more IP than white-feathered birds (Kjaer (2000), but see Uitdehaag, et al. (2008)). In our study, Brown birds indeed performed more IP than LSL birds (on average, 16 and 5 IP given, respectively). Despite selecting the birds pecking the most and the least for each strain, the lower overall levels of IP in LSL birds may have limited our ability to detect clear behavioral differences between P+ and P− individuals. Given these limitations, we will focus our discussion on the behavioral profile found in Brown birds, as the model may not have captured reliable variables for LSL birds.

**In Brown birds**, P+ birds appeared more socially motivated and less fearful than P- birds, adopting an overall more cautious approach of their environment. This was reflected by the greater amount of time they spent in the zone close to the conspecifics during the multivariate test, their shorter duration of tonic immobility, and the higher latencies to approach stimuli during the cognitive bias test. Pecking behavior is frequently associated with higher impulsivity, as demonstrated in behavioral studies investigating the serotoninergic system (de Haas and van der Eijk, 2018; Kops et al., 2017; van Hierden et al., 2004) or motor impulsiveness (Pichová et al., 2021). However, other studies have found contrasting results, with birds selected on their propensity to peck showing lower levels of recurrent perseveration (Kjaer et al., 2015) or inhibiting their motor response in a Go/No-Go task (Heinsius et al., 2020). Our results seem to support this, as the higher latency to approach the different stimuli in the cognitive bias test is more likely to reflect lower impulsivity, with P+ birds assessing their environment before getting into motion.

Lower fearfulness may also be reflected by the higher expression of comfort behavior in the home pen, as these behaviors are indicators of good welfare and expressed when the birds are relaxed in their environment (Boissy et al., 2007). These findings align with previous studies, as individuals from high pecking lines often appeared to be less fearful than individuals from low pecking lines (de Haas et al., 2010; van der Eijk et al., 2018), although variations between breeds are observed (Uitdehaag et al., 2008). Additionally, stronger social motivation in high peckers has been reported by Rodenburg and Koene (2003). In their study, hens from strains selected on their high propensity to peck vocalized more in a novel environment test than the one from low pecking strains, which can be interpreted as a stronger motivation to reinstate social contact (Forkman et al., 2007).

Also, contrary to our expectations, P+ birds expressed lower levels of foraging behavior. Injurious pecking is often linked to limited access to high-quality foraging substrate (de Haas et al., 2014b). In our study, birds had constant access to wood shavings, as the pens were lined with this substrate. One possible explanation could be the birds’ lack of interest for this specific type of substrate, leading them to redirect their foraging and exploratory behaviors towards conspecifics, resulting in higher levels of injurious pecking. However, a longitudinal study by Newberry, et al. (2007) found that birds that spent the most time foraging during the pullet stage were also those that pecked the most during adulthood, challenging the redirected foraging hypothesis. Alternatively, the low levels of foraging behavior might be explained by the fact that, if more socially motivated and engaging more with conspecifics, P+ birds have less time to engage in foraging activities. It is also important to note that we manipulated light intensity to increase levels of injurious pecking, and that response to this stressor might not necessarily be linked to foraging behavior.

These inconsistencies with previous literature may be related to differences in the birds’ genetic background. Indeed, as we show with our analysis, strains differed in their behavioral profile, and the sPLS-DA led on each strain did not identify behavioral profiles with the same reliability. In many experimental studies related to IP, authors used birds from high and low feather pecking lines (HFP and LFP). These two lines were selected from White Leghorns (Johnsen and Vestergaard, 1996), and not all behavioral indicators of IP found in these lines might be generalizable to other strains. Furthermore, most experimental studies focused on a subcomponent of IP, which is severe feather pecking. Behavioral traits associated with severe feather pecking might not be necessarily the same as the one associated with, for example, aggressive or vent pecking, even though several studies have reported positive correlations between severe feather pecking and aggressive pecking (Bennewitz, et al. (2014); Daigle, et al. (2015); Iffland, et al. (2021), but see Bessei, et al. (2013)).

Regarding practical application, these results could provide some insights for flock management. Adapting short behavioral tests for on-farm conditions or monitoring the whole flock behavior could potentially help to detect early signs of IP, before the apparition of plumage damage. However, this study was conducted in an experimental set up, and the practical implications of these findings should therefore be considered with caution. This study represents a first step towards a better understanding of the behavioral profile of injurious peckers, but these results need to be validated in larger social groups and under commercial production conditions. In the longer term, such knowledge could contribute to the development of approaches for identifying flocks at risk of developing IP, thereby allowing producers to monitor them more closely and, for example, provide additional enrichment to redirect pecking behavior at an early stage.

In conclusion, our study explored injurious pecking from a predictive perspective and identified behavioral profiles that may help anticipate injurious pecking outbreaks. However, the relatively high error rate observed in the analysis led on LSL birds calls for caution in generalizing these findings. It also underscores the importance of considering breed and strain differences when studying IP, and, more broadly, welfare related topics. Further research is still needed to validate the reliability of these behavioral indicators in larger flocks and across a wider range of breeds.

## Funding

This research was financially supported by the CASDAR fund (PeckLess project, 2023–2026).

## Declaration of generative AI and AI-assisted technologies in the writing process

During the preparation of this work the authors used ChatGPT in order to improve readability and language. After using this tool, the authors reviewed and edited the content as needed and take full responsibility for the content of the publication.

## Author contribution

**Lucille Dumontier** contributed to the conception and design of the study, performed the experiment, analyzed and interpreted the data, and wrote the first draft of the manuscript. **Vitor H. B. Ferreira** contributed to the conception and design of the study, reviewed and edited the manuscript. **Gaëlle Lefort** participated in data analysis. **Auriane Foreau, Julie Lemarchand** and **Fabien Cornilleau** contributed to the acquisition of data. **Maxime Quentin, Vanessa Guesdon, Maryse Guinebretière** and **Aude Kleiber** gave substantial inputs into the conception and design of the study. **Ludovic Calandreau** contributed to the conception and design of the study, reviewed and edited the manuscript.

All authors critically reviewed the manuscript, approved the final version, and take full responsibility for all aspects of the work presented.

## Disclosures

The authors declare that they have no known competing financial interests or personal relationships that could have appeared to influence the work reported in this paper.
