# Supplementary material for: Early behavioral predictors of injurious pecking: exploration of behavioral profiles in two commercial strains of laying hens
Source: Poult Sci. 2026 Oct 20;105(12):107998. doi: 10.1016/j.psj.2026.107998 (PMC13718418; doi:10.1016/j.psj.2026.107998)
Supplement: Supplementary file 1 [file mmc1.docx]

**Supp. 1:** Table showing the variable names for each test and a short description. For detailed description, see the complete ethograms and methods in the paper.

|  | Variable name | |  |
| --- | --- | --- | --- |
|  | Battery 1 | Battery 2 | Description |
| Detour test | Lat_Detour_1 | Lat_Detour_2 | Latency to perform the detour. |
| Novel object test | NO_prop.Z1_1 | NO_prop.Z1_2 | Proportion of scans spent in the zone close to the novel object. |
| Multivariate test | Zone1_1 | Zone1_2 | Time spent in the central zone. |
|  | Zone2_1 | Zone2_2 | Time spent in the outer zone. |
|  | Zone3_1 | Zone3_2 | Time spent behind the screens. |
|  | Lat_Z2_1 | Lat_Z2_2 | Latency to enter the outer zone. |
|  | Lat_Z3_1 | Lat_Z3_2 | Latency to enter one of the zones behind the screens. |
|  | Foraging_1 | Foraging_2 | Time spent foraging. |
|  | Transition_1 | Transition_2 | Number of transitions between the zones. |
| Cognitive bias test | Latency_Mirror_1 | Latency_Mirror_2 | Latency to approach the mirror. |
|  | Latency_Chicken_1 | Latency_Chicken_2 | Latency to approach the hen picture. |
|  | Latency_Owl_1 | Latency_Owl_2 | Latency to approach the owl picture. |
|  | Latency_Morph_1 | Latency_Morph_2 | Latency to approach the ambiguous stimulus (morph image). |
| Tonic Immobility | Lat_Righting_1 | Lat_Righting_2 | Duration of tonic immobility. |
|  | Induction_1 | Induction_2 | Number of inductions. |
| Time Budget | TB_Alert_1 | TB_Alert_2 | Proportion of time spent in alert behaviour. |
|  | TB_Comfort_1 | TB_Comfort_2 | Proportion of time spent performing comfort behaviours. |
|  | TB_Inactive_1 | TB_Inactive_2 | Proportion of time spent inactive. |
|  | TB_Nest_1 | TB_Nest_2 | Proportion of time spent in the nest. |
|  | TB_Forage_1 | TB_Forage_2 | Proportion of time spent foraging. |
|  | TB_Locomotion_1 | TB_Locomotion_2 | Proportion of time spent performing locomotory behaviours. |
|  | TB_Feeding_1 | TB_Feeding_2 | Proportion of time spent feeding/drinking. |

**Supp. 2:** Correlation matrix showing the correlation coefficients between all recorded variables. Darker colours reflect stronger correlations.

**Supp. 3:** Table showing the percentage of success for the detour test and cognitive bias test. Success was defined as the birds performing the detour or reaching the end of the corridor during the cognitive bias test.

|  |  |  | Brown | | | LSL | | |
| --- | --- | --- | --- | --- | --- | --- | --- | --- |
|  |  |  | All | P- | P+ | All | P- | P+ |
| Round 1 | Cognitive Bias | Mirror | 93 | 90 | 95 | 100 | 100 | 100 |
|  |  | Chicken | 91 | 95 | 85 | 96 | 82 | 100 |
|  |  | Owl | 73 | 71 | 65 | 40 | 53 | 40 |
|  |  | Morph | 91 | 95 | 90 | 90 | 88 | 90 |
|  | Detour | | 35 | 43 | 45 | 77 | 65 | 95 |
| Round 2 | Cognitive bias | Mirror | 94 | 100 | 85 | 99 | 94 | 100 |
|  |  | Chicken | 69 | 81 | 50 | 56 | 65 | 40 |
|  |  | Owl | 93 | 95 | 90 | 91 | 100 | 95 |
|  |  | Morph | 88 | 86 | 90 | 95 | 100 | 100 |
|  | Detour | | 64 | 62 | 55 | 91 | 88 | 90 |
